# Neoadjuvant chemoradiation therapy for borderline pancreatic adenocarcinoma: report of two cases

**DOI:** 10.1186/1477-7819-11-37

**Published:** 2013-02-05

**Authors:** José Galindo, Mauricio Gabrielli, Juan Francisco Guerra, Juan Carlos Cassina, Marcelo Garrido, Nicolás Jarufe, Yerko Borghero, Jorge Madrid, Pablo Zoroquiain, Juan Carlos Roa, Jorge Martínez

**Affiliations:** 1 Department of Digestive Surgery, Hospital Clínico Pontificia Universidad Católica de Chile, Marcoleta 350, Santiago, Chile; 2 Department of Hemato-Oncology, Hospital Clínico Pontificia Universidad Católica de Chile, Marcoleta 350, Santiago, Chile; 3 Department of Pathology, Hospital Clínico Pontificia Universidad Católica de Chile, Marcoleta 350, Santiago, Chile

**Keywords:** Borderline resectable pancreatic cancer, Neoadjuvant chemoradiation

## Abstract

Pancreatic cancer remains as one of the most aggressive human neoplasms, with overall poor survival rates. Radical surgery of the primary lesion is the best option for treatment. Borderline resectable pancreatic tumors (BRPT), defined as partial involvement of peripancreatic vasculature, may benefit from neoadjuvant therapy. We report on the first two BRPT cases treated with neoadjuvant chemoradiation at our institution. Preoperative CT and MRI demonstrated pancreatic tumors encasing the porto-mesenteric confluence suggestive of BRPT. Patients received neoadjuvant chemotherapy (gemcitabine/cisplatin), followed by radiochemotherapy. After treatment, follow-up images demonstrated tumor downsize, allowing for the tumors to be considered then as resectable. They underwent partial pancreatoduodenectomies (Whipple procedure). In case 1, histopathology revealed a complete, margin-free resection, whereas in case 2 there was a complete pathological response, with no evidence of residual tumor. According to the literature, our initial experience using neoadjuvant chemoradiotherapy on BRPT allowed us to downsize the tumor and, subsequently, to perform a curative surgery.

## Background

Pancreatic adenocarcinoma remains as one of the most devastating human cancers, with an overall 5-year survival of <5% [1]. Most patients are diagnosed with advanced disease and only 10 to 20% of patients are resectable at the time of diagnosis [2]. Radical surgery (R0 resection) of the primary tumor and regional lymph node dissection offers the only chance of long-term survival, but unfortunately the majority of those patients treated with curative surgical resection will eventually recur regionally or develop distant metastases [3,4].

Pancreatic tumors can be classified as resectable (stage I or II), locally advanced (stage III), or metastatic (stage IV). However, with recent advances in pancreatic imaging and surgical techniques, a distinct subset of tumors is emerging that blurs the distinction between resectable and locally advanced disease: tumors of ‘borderline resectability’ [5]. This group includes tumors that exhibit encasement of a short segment of the hepatic artery, without evidence of tumor extension to the celiac axis (CA) and tumor abutment of the superior mesenteric artery (SMA) involving <180° of the circumference of the artery; or short-segment occlusion of the superior mesenteric vein (SMV), portal vein (PV), or their confluence [5]. There is no consensus regarding the management of borderline resectable pancreatic tumors (BRPT). Several studies have suggested that the margin resection status is a very important prognostic factor and that a margin-positive resection strongly predicts early recurrence and short survival [5-8]. Neoadjuvant therapy of BRPT potentially can increase the likelihood of a margin-free resection. Also, it can serve as a biological marker of response to treatment [6]. We report our first two BRPT cases treated initially with neoadjuvant chemoradiation and subsequent radical surgery.

## Case presentation

### Case 1

**Figure 1:**
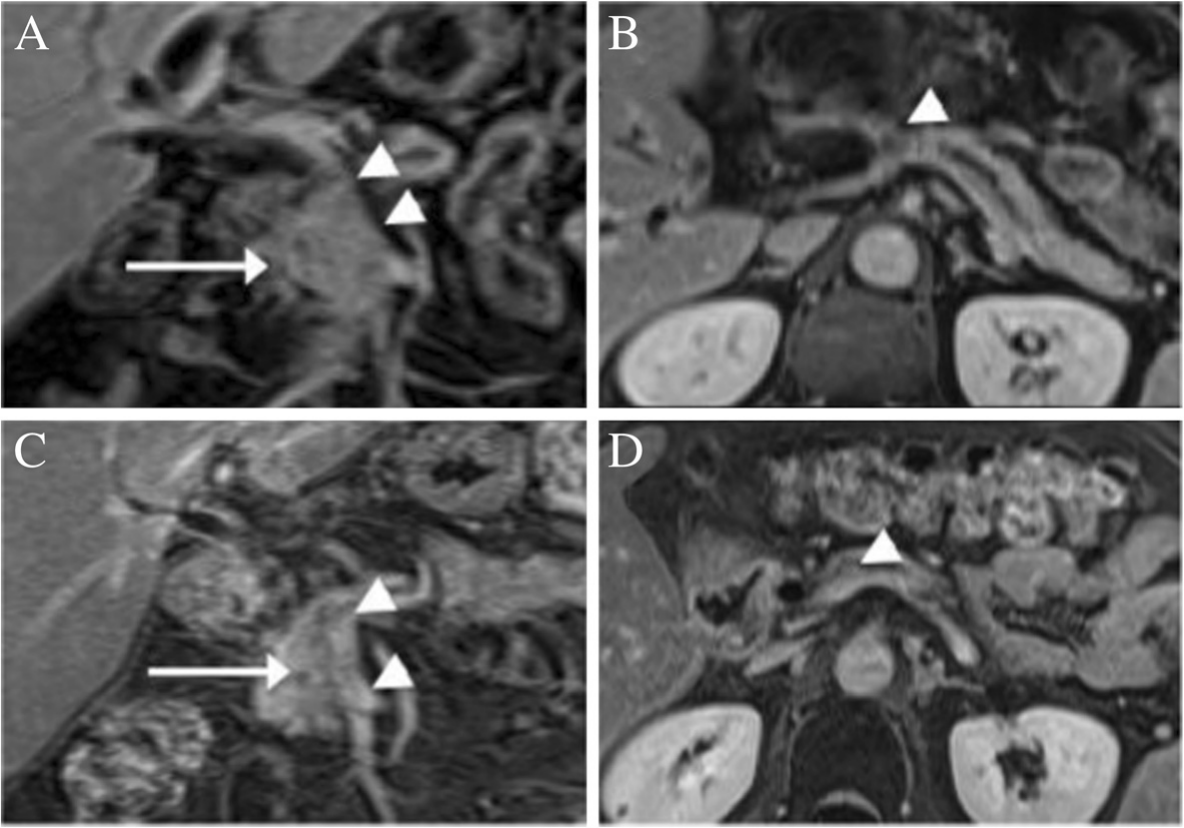
**MRI of solid tumor at the pancreatic neck in Case 1.** (**A**,**B**) Initial MRI, showing a hypovascular pancreatic tumor compatible with adenocarcinoma (arrow). The tumor is encasing the porto-mesenteric confluence (arrowheads); (**C**,**D**) MRI after neoadjuvant therapy. It shows a regression in the axial diameter of the tumor (arrow) and patency of the PV and SMV (arrowheads).

A 58-year-old man presented with weight loss, jaundice and abdominal pain. Laboratory parameters were compatible with obstructive jaundice. CA19-9 was elevated (214 UI/mL). MRI demonstrated a solid tumor at the pancreatic neck. The axial diameter of the tumor was 2.2 cm, encasing the porto-splenic-mesenteric confluence (>180°), with SMV compromise of 18 mm (longitudinal axis) and without compromise of CA and SMA (Figure 1A,B). There was no evidence of lymph node involvement or metastatic disease. An endoscopic ultrasound-guided fine needle aspiration biopsy was obtained. Pathology revealed a well-differentiated pancreatic adenocarcinoma. At the same time a biliary stent was placed. The patient received three cycles of chemotherapy (gemcitabine: 1,000 mg/m^2^ and cisplatin: 50 mg/m^2^) every two weeks. He also commenced radiotherapy (50.4 Gy in 30 fractions) and infusional 5-fluorouracil (200 mg/m^2^). The dose had to be reduced by 20% due to thrombocytopenia. At the end of treatment, CA19-9 dropped to 124 UI/mL. Follow-up MRI demonstrated further reduction in local disease, with no evidence of porto-mesenteric infiltration (Figure 1C,D). Based on his remarkable response the decision was made to explore the patient, so he underwent a Whipple procedure with curative intention.

**Figure 2:**
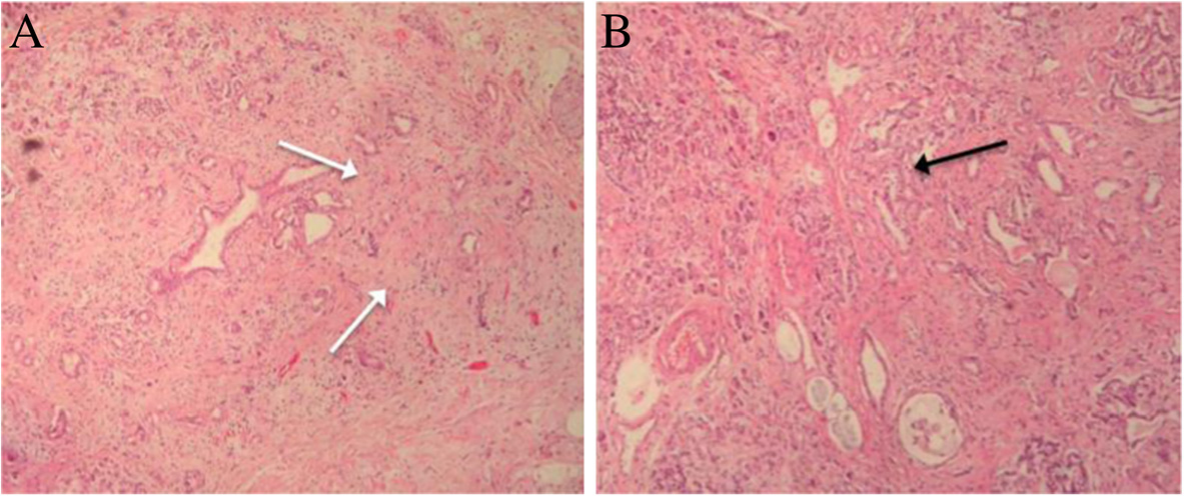
**Histopathology review. (A**) Pancreatic epithelium infiltrated by adenocarcinoma with large signs of regression (white arrows) (60x); (**B**) Tumor regression, which consists in multiple microscopic foci of infiltration into the macroscopic nodular area (black arrow) (100x).

Histopathological analysis of the specimen demonstrated a 1.5 cm, moderately differentiated pancreatic adenocarcinoma with extensive signs of regression, with multiple microscopic infiltrative foci (Figure 2). Surgical margins were free of disease.

### Case 2

**Figure 3:**
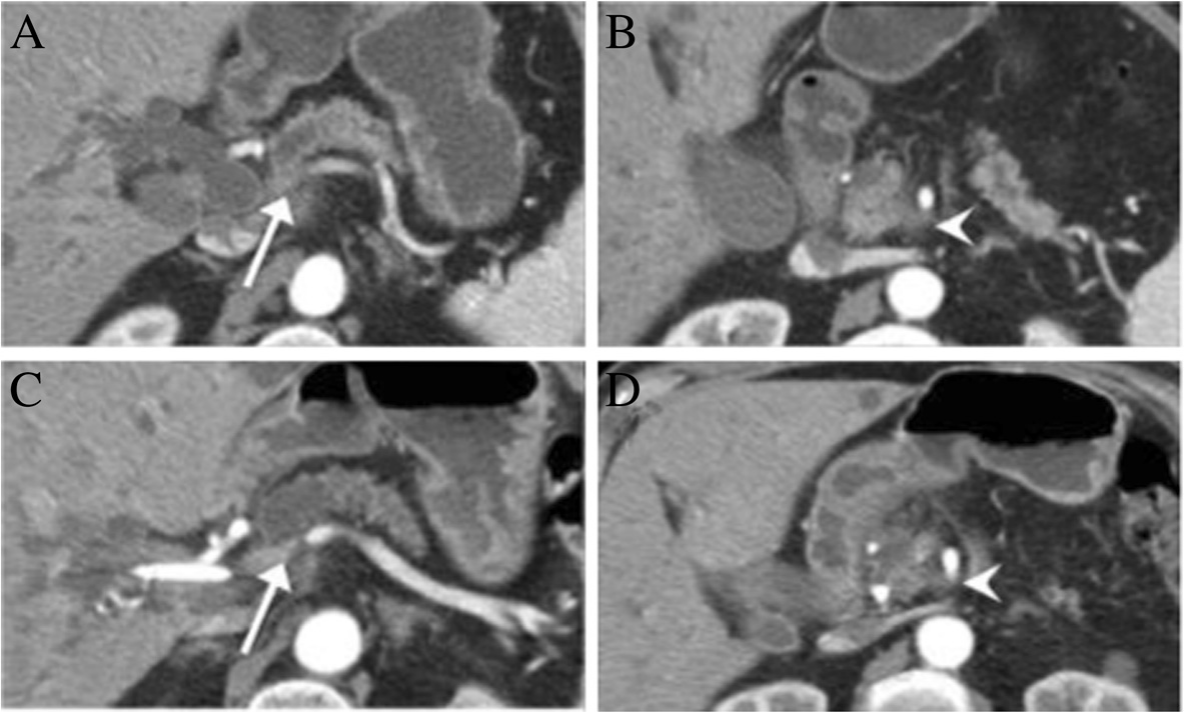
**Abdominal CT images for Case 2.** (**A**,**B**) Abdominal CT at the time of diagnosis, showing a pancreatic node compatible with a pancreatic tumor encasing the porto-splenic confluence (arrow) and attachment to the SMA (arrowhead); (**C**,**D**) Control CT after neoadjuvant therapy shows local disease reduction, with increased permeability of the PV (arrow) and less compromise of SMA (arrowhead).

A 72-year-old man presented with a history of abdominal pain, jaundice and weight loss. Laboratory results demonstrated a cholestatic pattern. CA19-9 was 37.8 UI/mL. Diagnostic studies revealed a 1.8 cm pancreatic mass, encasing the porto-splenic confluence (>180°) with attachment to the SMA (<180°) (Figure 3A,B). Unfortunately, it was not possible to perform a needle biopsy due to technical difficulties. However, images were analyzed by a group of expert radiologists and evaluated with a multidisciplinary team composed of oncologists, gastroenterologists and HPB surgeons, who agreed in the diagnosis of pancreatic cancer.

A biliary stent was placed, and relief of jaundice was achieved. He then commenced chemotherapy with gemcitabine (1,000 gm/m^2^) in combination with cisplatin (50 mg/m^2^) every two weeks. The patient completed three cycles of chemotherapy. During chemotherapy, cross sectional imaging suggested partial tumor regression, with increased patency of PV (compromise <180°) and less compromise of SMA (<90°), with no evidence of metastatic disease. The patient was referred for consolidation radiotherapy, of which he had a total of 50.4 Gy in 30 fractions and infusional 5-fluorouracil (200 mg/m^2^). He developed no major adverse effects.

Follow-up CT demonstrated reduction in local disease (Figure 3C,D). Eleven months after the initial diagnosis the patient underwent surgery with curative intent in the form of a Whipple procedure. He had an uneventful recovery period.

**Figure 4:**
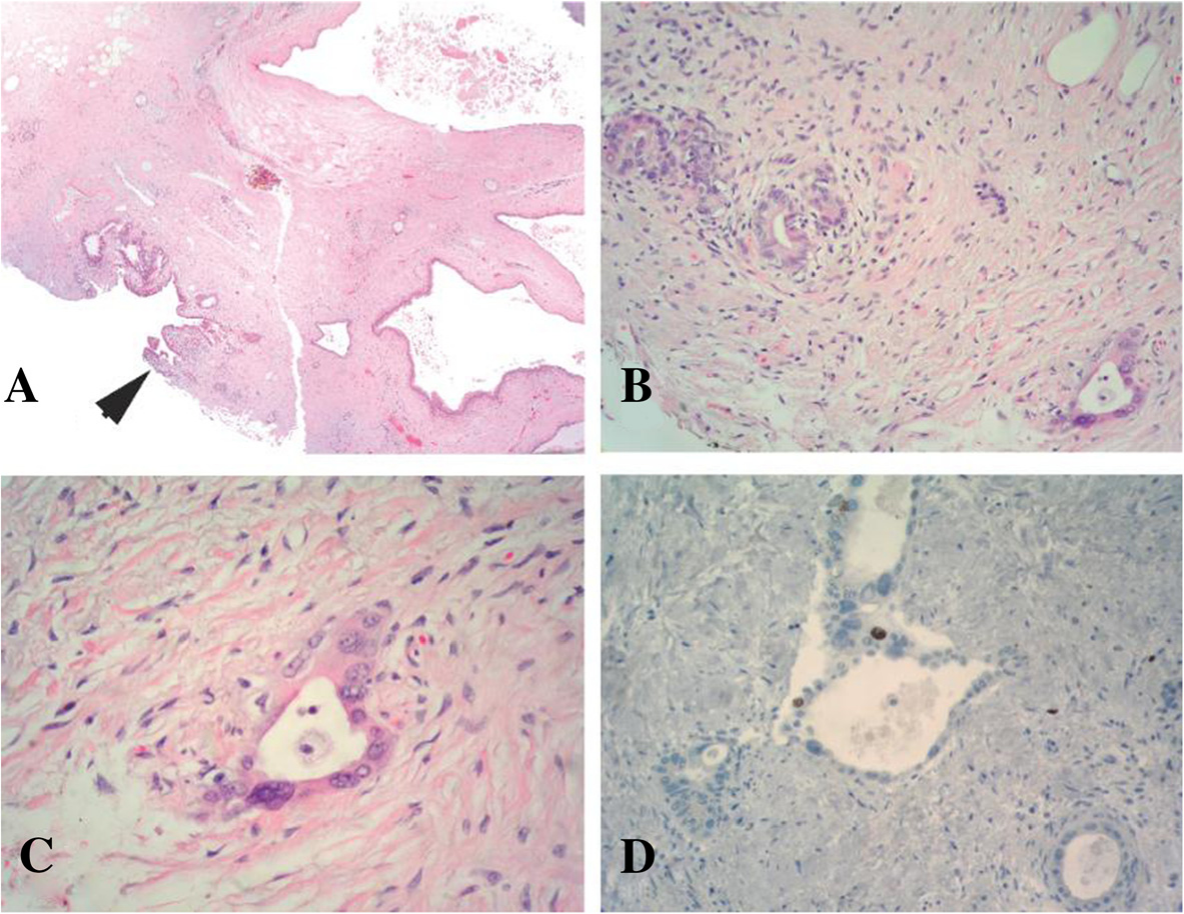
**Immunohistochemical analysis of Case 2.** (**A**) At low power, dilated pancreatic duct with normal epithelium is seen. At the lower left corner a pancreatic lesion with a few atypical secondary ducts is observed (arrow head); (**B**) At medium power, a few atypical ducts with a desmoplastic-like surrounding stroma is seen; (**C**) At high power, ducts are lined by irregular epithelium, with micronucleoli and irregular chromatin cells nuclei, suggestive but not consistent with carcinoma; (**D**) Ki-67 labeling shows nuclear reaction in less than 1% of the cells.

The whole pancreatic specimen was sent for pathological examination, processed in blocks of 3 to 4 mm thickness. Macroscopically, there was no evidence of tumor. Sixteen paraffin blocks, 18 slides and 19 sections were reviewed. Further immunohistochemistry failed to confirm a pancreatic carcinoma. These findings were considered as a complete pathological response (Figure 4).

## Discussion

Pancreatic cancer is a devastating malignancy with a 5-year overall survival of about 4% [1]. Margin-free resection of the tumor is considered the only chance of cure for these patients, although only 10 to 20% of the tumors are deemed to be resectable at the time of diagnosis. For most patients, curative surgery is not possible because of systemic metastases, advanced nodal disease or a localized tumor that is not amenable of resection due to invasion of adjacent vital structures [2].

Recently, the designation of borderline resectable tumors has emerged to describe a subpopulation of potentially resectable tumors. For tumors of the head or uncinate process, these criteria include SMV/PV compromise, SMA abutment, encasement of the gastroduodenal artery up to its origin at the hepatic artery, limited inferior vena cava involvement, short-segment SMV occlusion, and colon or mesocolon invasion [5].

Patients with borderline resectable tumors treated with surgical resection alone can be expected to have a higher rate of local and systemic disease recurrence and worse survival compared with patients who presented with initially resectable disease. This may be related to technical aspects (more difficult surgery), the advanced nature of the tumor, and the high risk for margin-positive resection. Therefore, the goal in the management of the borderline resectable patient is to maximize the chance of a complete resection, which may be accomplished by the use of neoadjuvant therapy [7].

The role of neoadjuvant therapy in borderline resectable disease is a highly debated topic [8,9]. This modality of treatment may allow tumor downsizing, reduce the incidence of positive resection margins, delivery of treatment to intact well-vascularized tissues, and higher rates of treatment completion. Also, it facilitates selection for surgery of patients with favorable tumor behavior. Patients who do not develop progressive disease prior to rescue surgery or patients with significant downsize response may have a better prognosis, and moreover, those with poor tumor biology are selected out via disease progression, thereby avoiding the morbidity of futile surgery [10].

Katz *et al.* reported on a group of patients with borderline resectable pancreatic adenocarcinoma treated with neoadjuvant chemoradiotherapy; 125 patients received this modality of treatment. Of these, 66 (41%) underwent pancreatectomy. Negative margin was obtained in 94% of the cases. Median survival was 40 months for patients who completed all therapy and 13 months for patients who did not undergo surgery [11]. McClaine *et al.* reported a 46% rate of surgical resection in a cohort of 26 patients with borderline pancreatic adenocarcinoma who underwent neoadjuvant chemoradiotherapy; 67% of them had a margin free resection. Median survival for resected patients was 23.3 months *vs.* 15.5 months for non-resected cases [12]. These two studies included a different number of patients; however, the difference was statistically significant in both. In another study, Brown *et al.* reported a cohort of 13 patients with borderline pancreatic adenocarcinoma who received neoadjuvant therapy. In 11 of 13 patients a margin-negative resection was achieved and nine patients were alive at 20 months follow-up [13]. A systematic review analyzed the role of neoadjuvant chemoradiotherapy for the treatment of both resectable and initially labeled as unresectable pancreatic cancer [14]. This study demonstrated that patients with unresectable pancreatic cancer who underwent neoadjuvant chemoradiotherapy achieved comparable 1-year survival as those with initially resectable disease; 40% of borderline or unresectable cases were ultimately resected. Also, it was not associated with a statistically significant increase in the rate of pancreatic fistula or overall complications in the chemoradiation group.

## Conclusions

Our initial experience in these two cases with borderline resectable tumors treated with neoadjuvant chemoradiation is encouraging. We recognize that the decision to offer this modality of treatment not having a biopsy suggestive of pancreatic adenocarcinoma (Case 2) might be debatable, but at the same time we believe that its radiological response made a neoplasm the most likely diagnosis, considering that other etiologies, such as chronic pancreatitis, should not be affected by chemoradiation. In any case, these results are consistent with the published literature, suggesting a clear benefit in terms of curative surgical resection. At this time our center is actively enrolling more patients to be considered for this modality of treatment.

## Consent

Written informed consents were obtained from the patients for publication of these cases reports and any accompanying images. Copies of the written consents are available for review by the editor-in-chief of this journal.

This research has been performed with the approval of ethics committee of our institution.

## Abbreviations

BRPT: Borderline resectable pancreatic tumors; CA: Celiac axis; SMA: Superior mesenteric artery; SMV: Superior mesenteric vein; PV: Portal vein.

## Competing interests

The authors declare that they have no competing interests.

## Authors’ contributions

JG, JFG, MG and JCC collected data and drafted the manuscript; JFG, NJ and JM participated in the design of the study and revised the manuscript for intellectual content; MG, JM and YB participated in the design of the study and chemoradiation protocol; PZ and JCR contributed to the histopathological section of the manuscript. All authors read and approved the final manuscript.
